# A Novel Automated Immunoassay Platform to Evaluate the Association of Adiponectin and Leptin Levels with Breast Cancer Risk

**DOI:** 10.3390/cancers13133303

**Published:** 2021-06-30

**Authors:** Debora Macis, Valentina Aristarco, Harriet Johansson, Aliana Guerrieri-Gonzaga, Sara Raimondi, Matteo Lazzeroni, Ivana Sestak, Jack Cuzick, Andrea DeCensi, Bernardo Bonanni, Sara Gandini

**Affiliations:** 1 Division of Cancer Prevention and Genetics, IEO, European Institute of Oncology IRCCS, 20141 Milan, Italy; valentina.aristarco@ieo.it (V.A.); harriet.johansson@ieo.it (H.J.); aliana.guerrierigonzaga@ieo.it (A.G.-G.); matteo.lazzeroni@ieo.it (M.L.); bernardo.bonanni@ieo.it (B.B.); 2 Molecular and Pharmaco-Epidemiology Unit, Department of Experimental Oncology, IEO, European Institute of Oncology IRCCS, 20141 Milan, Italy; sara.raimondi@ieo.it (S.R.); sara.gandini@ieo.it (S.G.); 3 Centre for Cancer Prevention, Wolfson Institute of Preventive Medicine, Queen Mary University of London, London EC1M-6BQ, UK; i.sestak@qmul.ac.uk (I.S.); j.cuzick@qmul.ac.uk (J.C.); andrea.decensi@galliera.it (A.D.); 4 Division of Medical Oncology, Ente Ospedaliero Ospedali Galliera, 16128 Genoa, Italy

**Keywords:** adiponectin, leptin, immunoassay platform, ELLA, ELISA, breast cancer risk

## Abstract

**Simple Summary:**

Adiponectin and leptin are adipokines secreted by the adipose tissue that have been associated with several chronic diseases including cancer. We compared two methods for their measurement and investigated their association with breast cancer. We measured adiponectin and leptin with the automated ELLA platform and a manual commercially available enzyme-linked immunosorbent assay (ELISA) kit on serum samples of women enrolled in two international breast cancer prevention trials. We found a good concordance between the two methods and our results support the association of low adiponectin levels with breast cancer, irrespective of the method used. The take-home message is that ELLA is a very robust platform that represents a step forward for the future use of adipokines, along with other biomarkers, in clinical cancer risk assessment and prevention. Its use should be taken into account whenever biomarkers should be measured in a large cohort of patients for clinical validation or cancer association studies.

**Abstract:**

Adiponectin and leptin are adipokines secreted by the adipose tissue that are associated with several chronic diseases including cancer. We aimed to compare the immunoassay platform ELLA with an enzyme-linked immunosorbent assay (ELISA) kit and to assess whether the results of the association analyses with breast cancer risk were dependent on the assay used. We measured adiponectin and leptin with ELLA and ELISA on baseline serum samples of 116 Italian postmenopausal women enrolled in two international breast cancer prevention trials. Results were compared with Deming, Passing–Bablok regression and Bland–Altman plots. Disease-free survival was analyzed with the Cox model. There was a good correlation between the methods for adiponectin and leptin (r > 0.96). We found an increased breast cancer risk for very low adiponectin levels (HR for ELLA = 3.75; 95% CI: 1.37;10.25, *p* = 0.01), whereas no significant association was found for leptin levels. The disease-free survival curves were almost identical for values obtained with the two methods, for both biomarkers. The ELLA platform showed a good concordance with ELISA for adiponectin and leptin measurements. Our results support the association of very low adiponectin levels with postmenopausal breast cancer risk, irrespective of the method used. The ELLA platform is a time-saving system with high reproducibility, therefore we recommend its use for biomarker assessment.

## 1. Introduction

Obesity is a huge burden worldwide, and its globally increasing prevalence in the last decades has reached epidemic proportions [1].

It is a major public health problem, since overweight and obese subjects are at a high risk of chronic diseases and multiple co-morbidities including diabetes, cardiovascular diseases and cancer [2]. A substantial loss of disease-free years associated with obesity is evident among men and women, irrespective of lifestyle factors [3].

Since the discovery of leptin in 1994 [4], it became apparent that the adipose tissue is not only an energy storage site, but a complex and highly active endocrine organ that regulates metabolic processes through the secretion of adipokines [5].

Leptin controls food intake and energy expenditure with a main function in the long-term regulation of appetite [6]. It is a pro-inflammatory adipokine and it has been suggested to mediate obesity-associated risk of cardiovascular diseases and to promote cancer through its actions in enhancing cell proliferation, reducing apoptosis and promoting migration and angiogenesis [7].

Adiponectin is an insulin-sensitizing hormone with anti-inflammatory, anti-atherogenic, antiangiogenic and antidiabetic properties. Low levels of circulating adiponectin have been associated with a variety of chronic diseases including type 2 diabetes, metabolic syndrome, coronary artery disease, polycystic ovarian syndrome, depressive disorders and cancer [6,7].

In the last decade, our group has focused on the association of adiponectin and leptin with breast cancer. Our results from original data [8,9] and meta-analysis [10] support the association of low adiponectin levels with increased risk of breast cancer.

Given the emerging role of adiponectin and leptin as risk biomarkers in cancer and other diseases, implementing laboratory methods could be useful for a personalized risk assessment.

Over the past few decades, different assays have been used, but a gold standard has not yet been established. One of the most common techniques is the enzyme-linked immunosorbent assay (ELISA), since many different kits are commercially available and easy to perform. However, ELISA kits require a long time span and the performance is subject to intra- and inter-assay variation by the operator and other analytical errors [11].

The aim of the present work was to compare adiponectin and leptin values obtained with a commercially available ELISA kit with those obtained with a new automated platform named ‘ELLA’. The comparison between the two methods was performed on healthy postmenopausal women at increased breast cancer risk due to age, family history, reproductive characteristics or high mammographic density, and on postmenopausal women with a resected Ductal Carcinoma In Situ (DCIS) of the breast. Furthermore, we aimed to verify whether similar estimates of association of adiponectin and leptin levels with breast cancer could be obtained with both methods.

## 2. Materials and Methods

### 2.1. Participant Selection

We randomly selected baseline frozen serum samples from the Italian cohort of postmenopausal women participating in the International Breast Cancer Intervention Study (IBIS)-II Prevention (*n* = 186) and DCIS (*n* = 348) trials.

The IBIS-II Prevention study is an international, double-blind, placebo-controlled phase III trial in which 3864 healthy postmenopausal women at increased breast cancer risk were randomized to receive anastrozole 1 mg/day or matching placebo for 5 years (EudraCT n. 2004-00391-12) [12] and were followed up for an additional 10 years. Briefly, the increased breast cancer risk was evaluated as at least two-fold the relative risk for breast cancer in the general population based on age, family history of breast or ovarian cancer, reproductive history or a mammographic density above 50%.

The IBIS-II DCIS study is an international, double-blind, phase III trial in which 2980 postmenopausal women with a resected estrogen receptor positive DCIS of the breast were randomized to receive anastrozole 1 mg/day or tamoxifen 20 mg/day for 5 years (EudraCT n. 2004-003992-35) [13] and were followed up for an additional 10 years.

Further details and results of the IBIS-II trials have been published elsewhere [12,13,14].

The trials were approved by the UK North West Multicentre Research Ethics Committee and by the ethics committees of all participating institutions. All participants provided written informed consent. This research was conducted in accordance with the Declaration of Helsinki.

### 2.2. Sample Collection and Storage

Fasting blood samples were collected at baseline (before treatment) for subjects randomized in the IBIS-II trials. Serum separator tubes were allowed to clot for 30 min before centrifugation for 10 min at 1850× *g*. Serum was aliquoted and stored at −80 °C.

We randomly drew out baseline serum samples from 116 subjects for adiponectin measurement and from 104 subjects for leptin measurement, based on specimen availability. The two cohorts of subjects partially overlapped. We measured adiponectin and leptin with two different methods.

### 2.3. ELISA

We used the Quantikine ELISA kits (R&D Systems, Bio-techne, Minneapolis, MN, USA) described below.

The “Quantikine Human Total Adiponectin/Acrp30 Immunoassay” is a solid-phase ELISA designed to measure total (low, middle and high molecular weight) human Adiponectin. The lower limit of quantitation (LLOQ) is 3.9 ng/mL, the upper limit of quantitation (ULOQ) is 250 ng/mL and the limit of detection (LOD) is 0.891 ng/mL. The assay time is 4.5 h.

The “Quantikine Human Leptin Imunoassay” is a solid-phase ELISA designed to measure soluble human Leptin. The LLOQ is 15.6 pg/mL, the ULOQ is 1000 pg/mL and the LOD is 7.8 pg/mL. The assay time is 3.5 h.

The absorbance of ELISA plates was read with the Epoch^TM^ Microplate Spectrophotometer (BioTek Instruments, Inc., Winooski, VT, USA), controlled with the Gen5 Software interface.

Both ELISA assays required a 100-fold dilution factor for serum samples that we performed with a single-step dilution.

In each Quantikine ELISA plate we ran 35 samples and 3 levels of Quality Controls (QCs, low, medium and high level from the Quantikine Immunoassay Control Set for Human Adiponectin and Leptin, R&D Systems) in duplicate and one internal control (pool of sera) in quadruplicate. The intra-assay coefficient of variation (CV) was evaluated by analyzing serum samples. The inter-assay CV was calculated for QCs and pool. We strictly followed the manufacturer’s instructions.

### 2.4. ELLA

ELLA (ProteinSimple, Bio-techne, Minneapolis, MN, USA) is a platform based on a microfluidic technology that allows the performance of automated enzyme linked immunoassays without manual steps. The only steps that need operator handling are the dilution of samples and the loading of diluted samples and washing buffer into the cartridge. The calibration curve for each cartridge is generated by the manufacturer for each lot and the ELLA system acquires calibration-related parameters through the reading of the cartridge’s barcode. The fluorescent signals are read inside the ELLA instrument and used for quantification based on master calibration curves. Further details on the ELLA platform have been clearly described elsewhere [15,16]. The assay time is 72 min. The cartridges are available in a multi- or single-analyte format.

We used 72-plex single-analyte cartridges for human adiponectin and leptin.

The adiponectin assay requires a 2000-fold dilution factor that we performed with a 3-step dilution as recommended by the manufacturer (1:20; 1:10; 1:10) and with a 2-step dilution (1:40; 1:50). We transferred a volume of 10 µL in each step of the dilutions. The LLOQ is 20.7 pg/mL, the ULOQ is 51,540 pg/mL and the LOD is 6.24 pg/mL for the adiponectin assay.

The leptin assay requires a single-step 1:10 dilution. The LLOQ is 2.20 pg/mL, the ULOQ is 13,810 pg/mL and the LOD is 1.71 pg/mL for the leptin assay.

In each cartridge we ran 69 samples, two levels of QCs (low and high level, Bio-techne) and one internal control (pool of sera). Samples and controls were automatically performed in triplicate by the ELLA system. The intra-assay CV was evaluated by analyzing serum samples. The inter-assay CV was calculated for QCs and pool.

### 2.5. Statistical Analysis

We present descriptive statistics of age, body mass index (BMI), adiponectin and leptin values obtained with ELISA and ELLA by disease status.

We tested the normality assumption of variables by Shapiro Wilk test and found that biomarkers, BMI and age are not normally distributed (*p* < 0.0002). Thus, we used the non-parametric Wilcoxon rank sum test to compare median values in the two studies.

We calculated the Pearson’s r coefficient in order to measure the strength of the correlation between the two variables. However, it is not a robust technique for the evaluation of the agreement between them [17] and it cannot detect systematic biases but random error only [18].

We then performed Passing–Bablok [19] and Deming regression [20] analyses for methods comparison in order to estimate the constant and the proportional errors by the intercept and the slope of the regression lines, respectively. The Passing–Bablok regression is not affected by the distribution of samples and measurement errors or data outliers, but it does not take into account random differences between methods [21]. The Deming regression assumes that the measurements by both methods are subjected to random errors [22]. We evaluated the agreement between the two methods by way of Bland–Altman plots [23], investigating the correspondent 95% limits of agreement (LoA) of the mean difference [17]. We repeated the Bland–Altman analysis separately on low and high values of adiponectin and leptin, using as cut-offs the first and the third quartile for adiponectin and leptin, respectively, as for the following survival analysis (see below).

The Spearman correlation coefficient was also calculated to estimate relationships between the variables.

Disease-free survival (DFS) was calculated including subjects with resected DCIS and high risk healthy subjects. For both groups, the event of interest was a diagnosis of breast cancer or DCIS. DFS curves were estimated with the Kaplan–Meyer method and the independent association of leptin and adiponectin with breast events was investigated with Cox proportional hazard models. We present hazard ratios for breast cancer, adjusting for risk strata and treatment arms.

## 3. Results

### 3.1. Baseline Characteristics of Subjects

Table 1 shows baseline median and interquartile ranges (IQR) values of age, BMI, serum adiponectin and leptin levels measured with the two methods by disease status (healthy at increased risk subjects from the Prevention IBIS-II Trial versus subjects with resected DCIS from the DCIS IBIS-II Trial).

We did not observe statistically significant differences for any of the variables except for a slightly higher BMI in healthy compared to DCIS subjects.

### 3.2. Assay Precision of ELISA and ELLA Methods

Adiponectin and leptin were detectable in all samples using the ELISA and ELLA methods.

The adiponectin and leptin median values, IQRs and minimum and maximum values are reported in Table 2.

Considering replicates of the same sample, we obtained intra-assay CVs below 5% and inter-assay CVs below 10% with ELLA or ELISA methods for adiponectin. The intra-assay CVs for leptin were below 2% for both methods, whereas the inter-assay CVs were below 10% except for QCs assessed with the ELISA method that showed an inter-assay CV of 16.63% (Table 2).

### 3.3. Methods Comparison ELISA vs. ELLA

#### 3.3.1. Adiponectin

**Figure 1:**
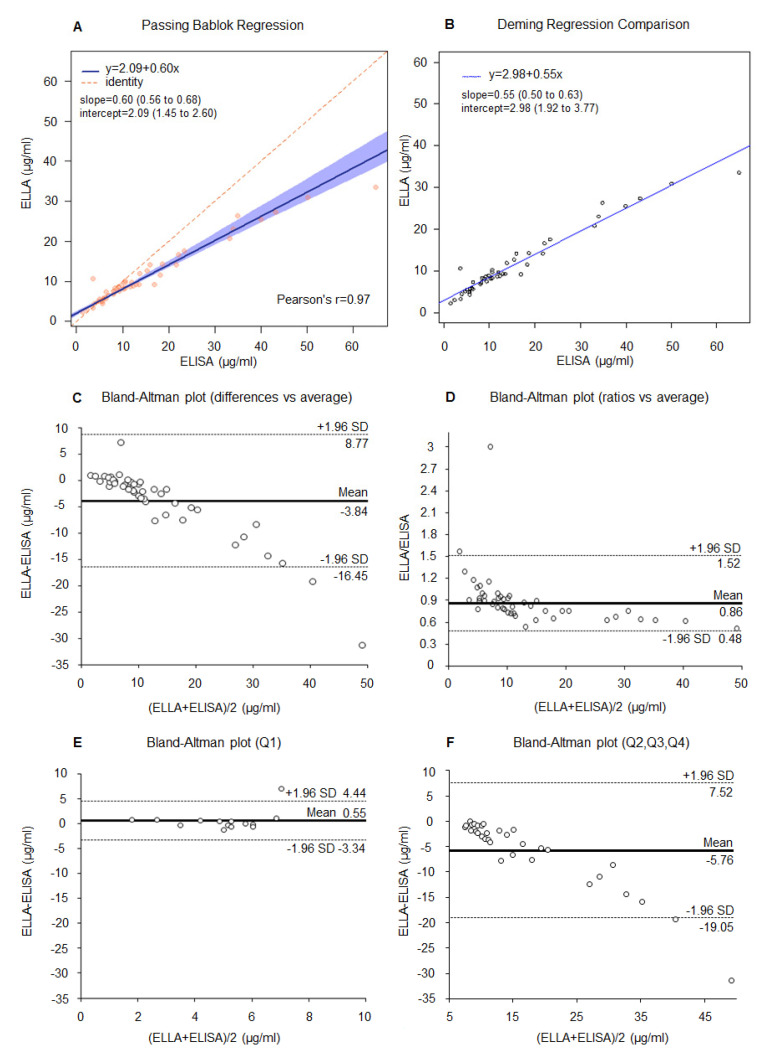
Correlation between ELLA (3-step dilution protocol) and ELISA for adiponectin measurement (*n* = 46). (**A**) Passing–Bablok and Pearson’s r coefficient, (**B**) Deming regression, (**C**) Bland–Altman plot (differences vs. average), (**D**) Bland–Altman plot (ratio vs. average), (**E**) Bland–Altman plot (differences vs. average) for adiponectin < 6.75 µg/mL (<first quartile Q1), and (**F**) for adiponectin ≥ 6.75 µg/mL (≥first quartile, Q2-3-4). Dashed lines represent 95% LoA.

We first compared adiponectin results obtained with the ELLA platform on 3-step diluted samples (*n* = 46) with results obtained with the ELISA method on the same samples (Figure 1).

The Pearson correlation coefficient r = 0.97 (Figure 1A) showed an overall good correlation between the ELISA and the ELLA methods with the 3-step dilution protocol. The Passing–Bablok and Deming regression lines are presented in Figure 1A,B, respectively. There is statistically significant evidence that, for the higher values of adiponectin obtained with the ELISA method, there were relatively lower values obtained with ELLA.

The Bland–Altman plot showed a mean difference of –3.84 µg/mL of adiponectin values measured with ELLA compared to ELISA (Figure 1C). The mean ELLA/ELISA ratio of 0.86 indicated that ELLA adiponectin values were on average 14% lower compared to ELISA (Figure 1D). Looking in depth at the differences between low and high adiponectin levels, the Bland–Altman plots highlighted no virtual difference between ELLA and ELISA for low adiponectin values and, on the other side, an underestimation for higher adiponectin values. Indeed, for adiponectin values lower than 6.75 µg/mL (first quartile of adiponectin measured with ELISA), ELLA adiponectin values were on average 0.55 µg/mL higher compared to ELISA (Figure 1E). On the contrary, for adiponectin values higher than 6.75 µg/mL, ELLA measurements were on average 5.76 µg/mL lower compared to ELISA, with the highest differences observed for higher adiponectin levels (Figure 1F).

**Figure 2:**
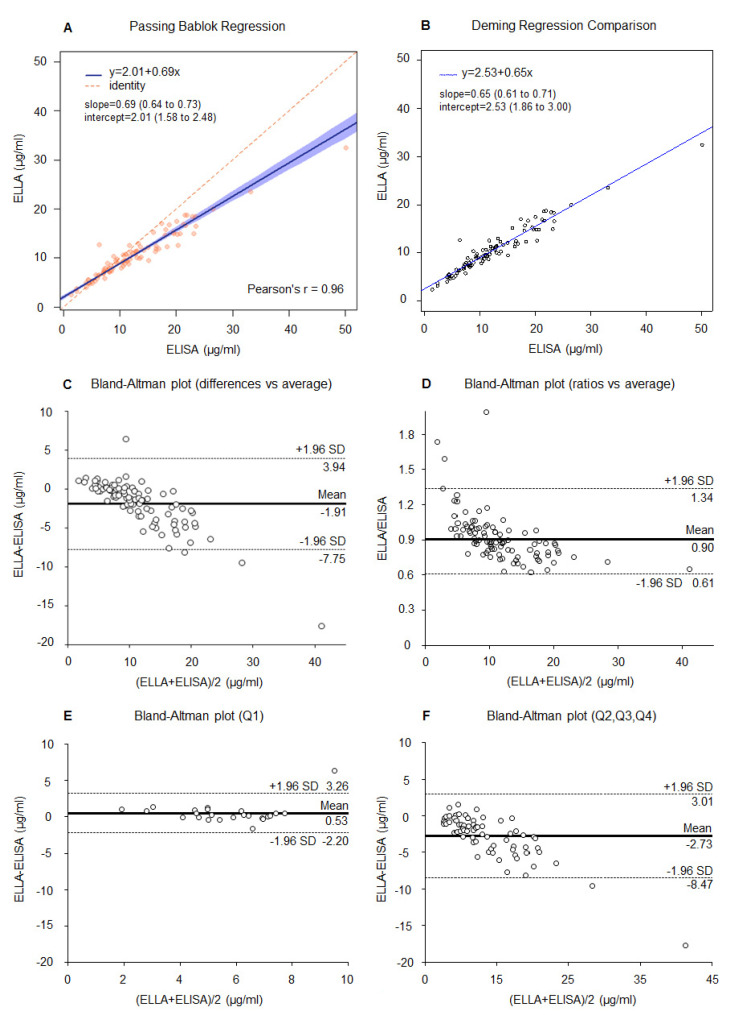
Correlation between ELLA (2-step dilution protocol) and ELISA for adiponectin measurement (*n* = 95). (**A**) Passing–Bablok and Pearson’s r coefficient, (**B**) Deming regression, (**C**) Bland–Altman plot (differences vs. average), (**D**) Bland–Altman plot (ratio vs. average), (**E**) Bland–Altman plot (differences vs. average) for adiponectin < 6.75 µg/mL (<first quartile Q1), and (**F**) for adiponectin ≥ 6.75 µg/mL (≥first quartile, Q2-3-4). Dashed lines represent 95% LoA.

We then compared adiponectin values measured with the ELLA platform on 2-step diluted samples (*n* = 95) with those measured with the ELISA method and the results were similar to the previous 3-step comparison. The Pearson correlation coefficient r = 0.96 (Figure 2A) again showed a good correlation between the two methods. The regression lines obtained with the Passing–Bablok and Deming regression analysis are presented in Figure 2A,B, respectively. These results confirm that, with increasing values of adiponectin, we have lower values with ELLA compared to ELISA, especially for high values of adiponectin. The Bland–Altman plots showed similar results to those previously described for the 3-step diluted samples, with a decreased width of the 95% LoA (Figure 2C–F).

#### 3.3.2. Leptin

**Figure 3:**
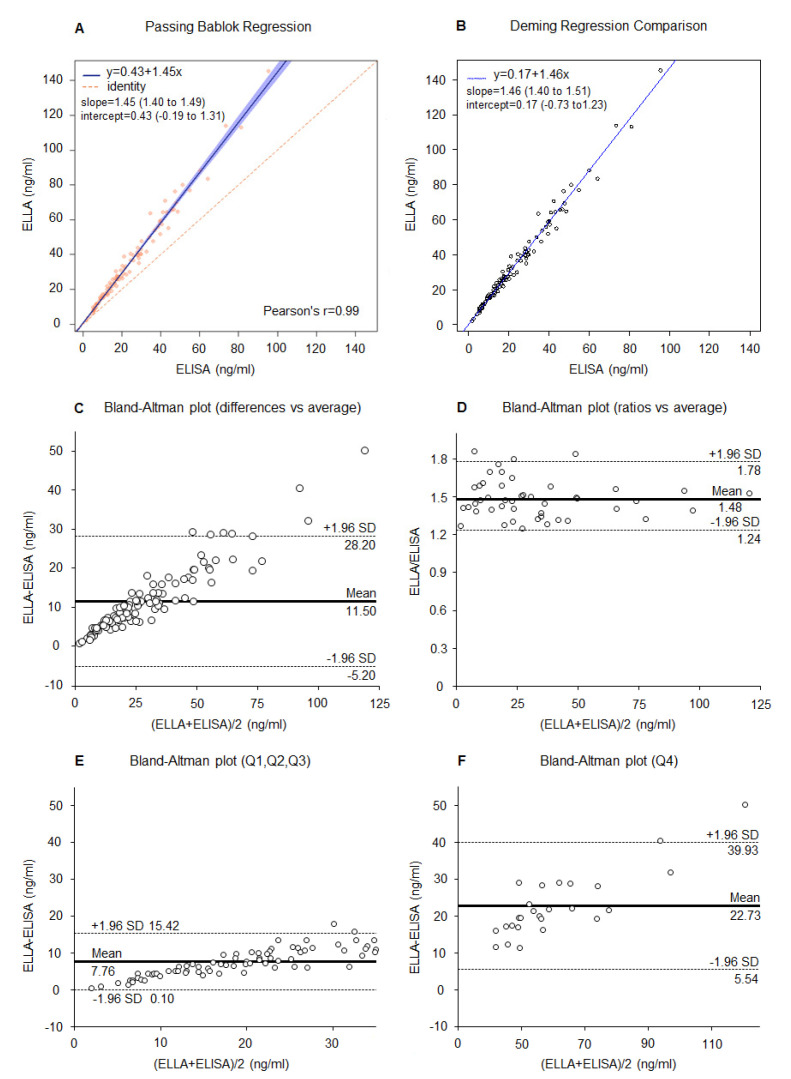
Correlation between ELLA and ELISA for leptin measurement (*n* = 104). (**A**) Passing–Bablok and Pearson’s r coefficient, (**B**) Deming regression, (**C**) Bland–Altman plot (differences vs. average), (**D**) Bland–Altman plot (ratio vs. average), (**E**) Bland–Altman plot (differences vs. average) for leptin < 33 ng/mL (<third quartile, Q1-2-3), and (**F**) for leptin ≥ 33 ng/mL (≥third quartile, Q4). Dashed lines represent 95% LoA.

The Pearson correlation coefficient r = 0.99 demonstrated an excellent agreement between the ELISA and the ELLA methods (Figure 3A). The Passing–Bablok and Deming regression lines are presented in Figure 3A,B, respectively. The overall Bland–Altman analysis highlighted a positive mean difference of 11.50 ng/mL with ELLA compared to ELISA (Figure 3C). The mean ratio of the ELLA/ELISA measures was 1.48 indicating an average overestimation of leptin values of 48% with ELLA compared to ELISA (Figure 3D). The Bland–Altman plots for lower and higher leptin levels demonstrated that ELLA measurements were 7.76 ng/mL higher for leptin and were lower than 33 ng/mL (third quartile when measured with the ELISA method, Figure 3E), and 22.73 ng/mL higher for leptin values higher than 33 ng/mL (Figure 3F).These results confirm the evidence that with increasing value of leptin we have greater values with ELLA compared to ELISA.

### 3.4. Association with Clinical Data by Method (ELISA vs. ELLA)

We did not find any association between adiponectin and leptin and age, measured by both methods, whereas a statistically significant correlation of adiponectin and leptin with BMI was observed (Spearman ρ = −0.25 (*p* = 0.008) for adiponectin measured with ELISA or ELLA and ρ = 0.72 (*p* < 0.0001) for leptin measured with ELISA or ELLA). Adiponectin and leptin were inversely correlated irrespective of the method used.

After a median follow-up of 8 years (absolute range: 2.6–13.8 years), 21 breast cancer events and one death were observed. The characteristics of breast cancers that occurred during the follow-up are reported in the Supplemental Table S1. Nine women who had breast cancer during follow-up were in the placebo groups and 12 women were in the intervention group (anastrozole or tamoxifen).

**Figure 4:**
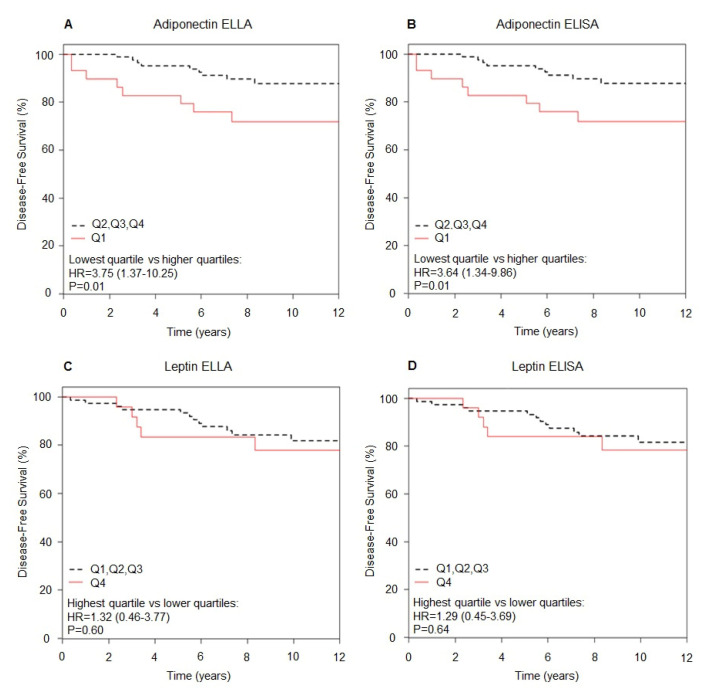
Disease-free survival curves according to lowest quartile versus higher for adiponectin (*n* = 116) measured with ELLA (**A**) or ELISA (**B**) and highest quartile versus lower for leptin (*n* = 104) measured with ELLA (**C**) or ELISA (**D**). Disease-free survival was calculated including subjects with resected DCIS and high risk healthy subjects.

Figure 4 shows the DFS curves for adiponectin and leptin measured with ELISA and ELLA methods. We found an increased breast cancer risk with very low adiponectin levels at baseline.

Considering adiponectin values measured with the ELLA method, we found an almost 4-fold increased breast cancer risk HR = 3.75 (95% CI: 1.37;10.25, *p* = 0.01) for subjects with low adiponectin values (below the lowest quartile) versus subjects with higher adiponectin (Figure 4A). We found a comparable risk estimate for adiponectin values measured with ELISA (HR = 3.64; 95% CI: 1.34;9.86, *p* = 0.01, Figure 4B).

No significant association between leptin values and breast cancer risk was observed considering neither values measured with the ELLA method (HR = 1.32; 95% CI: 0.46;3.77, highest quartile versus lower quartiles, *p* = 0.60, Figure 4C), nor with the ELISA method (HR = 1.29; 95% CI: 0.45;3.69, *p* = 0.64, Figure 4D).

The DFS curves for adiponectin and leptin measured with ELLA and ELISA methods by quartiles are shown in Supplemental Figure S1. There were small changes of the position of samples in their respective quartiles for each method, but this does not have a relevant impact on the DFS curves, which were very similar.

## 4. Discussion

This is the first study evaluating the use of the automated ELLA platform for serum adiponectin and leptin measurement. We compared the results obtained with the ELLA system to those obtained with the Quantikine ELISA kit from R&D Systems. Interestingly, we observed very similar risk estimates of adiponectin and leptin with DFS irrespective of the method used. Indeed, the almost four times higher risk of breast cancer for very low adiponectin levels compared to higher levels was found equally with both methods.

The intra- and inter-assay CVs indicated good within-run and between-run precision for both methods [24]. However, we found a high inter-assay CV for leptin QCs assessed with the ELISA method. This was probably due to a low stability of the lyophilized QCs after reconstitution, considering that our internal control indicated a good between-run precision also for the ELISA method with a CV < 10%.

The Pearson’s r coefficient for data obtained with the ELISA or ELLA methods were all above 0.95, reaching a value of 0.99 for leptin, indicating an excellent linear relationship between the two sets of data. We obtained similar regression lines with the Passing–Bablok and Deming regression methods for both adiponectin and leptin. The regression equations for adiponectin revealed the presence of proportional differences between the measurements with the two methods, irrespective of the sample dilution protocol used for the ELLA method (2-step or 3-step). However, we should consider that, given the high 2000-fold dilution factor recommended for samples in the adiponectin assay on the ELLA instrument, the 2-step dilution protocol allows a reduction of the additional time for sample preparation and errors in sample handling throughout dilution steps; nevertheless it requires higher extra volumes of dilution buffer to be specifically supplied by the manufacturer.

The regression equations for leptin showed similar results between the two methods.

The proportional differences observed for adiponectin and leptin between the two methods may be explained by differences at calibration levels [25]. The dynamic range of the ELLA method is much wider and the sensitivity higher.

Since the discovery of leptin and adiponectin, different methods have been utilized for their measurements. The most commonly used methods are the radioimmunoassay (RIA) and the ELISA. Despite the high sensitivity, the RIA technique has several disadvantages, mainly the hazard related to the use of radioactivity; it requires specifically trained personnel, a specific laboratory license for the handling, storage and waste disposal of radioactive material. Furthermore, it is expensive, time-consuming and it requires costly equipment. For all these reasons, researchers have tested alternative more feasible methods that can replace the RIA technique [25,26,27,28,29,30]. Among the methods, many authors evaluated the multiplexed beads immunoassay technology that allows the measurement of a large number of analytes with small sample volumes [27,29,30,31,32]. This technique should be considered when many different analytes related to a specific disease or condition could be combined in one panel, allowing a reduction of time and costs.

Given the evident involvement of adiponectin and leptin in metabolic processes and their association with many diseases, the measurement of these adipokines in large cohorts of subjects in epidemiological studies and clinical trials has become crucial to elucidate their role and correlations with clinical data. In the clinical context, the automation of laboratory testing appears to be relevant. Some authors have reported methods comparison studies testing adiponectin with immunoturbidimetry [33], chemiluminescent immunoassays [30] and ELISA performed on automatic routine clinical chemistry bioanalysers [28].

To date, this is the first study reporting the use of the ELLA platform for adiponectin and leptin measurements. Aldo et al. published an exhaustive paper explaining how the ELLA system works and presenting data for mouse and human cytokines and chemokines detection [16]. The authors highlighted the advantages of using the ELLA system in terms of required sample volumes, higher sensitivity and dynamic range, better coefficient of variation, and reproducibility compared to ELISA and the Luminex multiplex platform.

In the present work, one important result is that we found the same association with DFS irrespective of the method used for adiponectin and leptin measurement. Despite the limitations of our study, specifically the small sample size and different cohorts for adiponectin and leptin measurements, we confirmed a significant increased risk of breast cancer for subjects with very low adiponectin levels. This result is consistent with previous original data we published in premenopausal women [9] and meta-analysis results [10]. More recently, further meta-analyses have been published confirming the association between low adiponectin levels and higher breast cancer risk [34,35,36,37]. Interestingly, the latest research by Befort et al. reported that sustained increases in adiponectin after a 10% weight loss likely confer benefits for breast cancer prognosis even with weight regain [38].

On the contrary, data from meta-analyses showed an association between high leptin levels and higher breast cancer risk [35,36,39,40,41]. We observed a positive, although not significant, association between leptin and breast cancer, probably due to the small sample size and the weakness of the association.

Subgroup analyses from recent meta-analyses showed that leptin and adiponectin were strongly associated with breast cancer in Asians compared to non-Asians, in obese compared to non-obese subjects and postmenopausal compared to premenopausal women [35,37,39,41].

Interestingly, subgroup analyses by detection methods for adiponectin and leptin measurements showed that statistically significant differences were observed when ELISA or RIA were used [37,39,41], whereas the difference was not more significant upon detection by Multiplex assay [35] and in one meta-analysis, nor with RIA [34]. However, the observed differences could be due to the numerousness of studies analyzed.

In summary, we observed slightly higher measured values for low adiponectin levels and lower measured values for high adiponectin levels with ELLA compared to ELISA, whereas leptin measured values were systematically higher with ELLA compared to ELISA. Despite the observed differences, the overall agreement between methods was good. The most striking result was that the observed statistically significant association of adiponectin and leptin with DFS did not change by ELLA or ELISA detection methods. One limitation of our study is the small sample size. The extensive analysis we conducted to compare the two methods should surely be considered when pooling biomarker results from different studies becomes essential to increase the sample size.

Our results showed a significant negative association between adiponectin and breast cancer in a cohort of healthy postmenopausal women at increased risk for breast cancer or postmenopausal women with resected DCIS of the breast, further supporting the role of adiponectin in breast cancer. However, mechanisms and causal relationships still need to be elucidated. In addition, normal and pathological levels of leptin and adiponectin should be clearly defined, as their assessment could be useful to clinicians for a better definition of risk categories of cancer and other chronic conditions. Actually, further clinical studies are mandatory and the introduction of the automation of laboratory methods is valuable. The ELLA platform is an easy, automated system that allows the performance of immunoassays without manual steps by the operator and the processing of up to 70 samples for a single analyte in triplicate in a short time with high results reproducibility and a reduction of costs compared to ELISA.

## 5. Conclusions

In conclusion, our data suggest that ELLA is a very robust platform. It represents a step forward for the future use of adipokines, along with other biomarkers, in clinical cancer risk assessment and prevention.

## Figures and Tables

**Table 1:** Baseline characteristics of subjects.

	Subjects Randomized in the Prevention IBIS-II Trial				Subjects Randomized in the DCIS IBIS-II Trial				
Variable	*n*	Median	Lower Quartile	Upper Quartile	*n*	Median	Lower Quartile	Upper Quartile	*p*-Value ^1^
Adiponectin ELISA	63	11.7	8.6	16.9	53	10.6	6.4	18.0	0.391
Adiponectin ELLA	63	10.1	8.6	12.6	53	9.6	6.8	14.7	0.719
Leptin ELISA	53	21.2	13.5	30.2	51	17.4	9.1	36.1	0.314
Leptin ELLA	53	31.4	21.8	43.0	51	26.4	14.3	50.0	0.227
BMI	69	25.7	23.6	29.1	61	24.5	22.3	27.3	0.034
Age	69	59	55	64	62	59	53	63	0.829

^1^*p*-value for the difference between the two trials.

**Table 2:** Adiponectin and leptin values, intra- and inter-assay precision by method.

	Adiponectin		Leptin	
	ELLA	ELISA	ELLA	ELISA
Median (IQR ^1^)	9.96 µg/mL (7.39; 12.76)	11.28 µg/mL (7.73; 17.54)	28.59 ng/mL (17.48; 47.67)	19.66 ng/mL (12.28; 32.95)
Min; Max	2.23; 33.50 µg/mL	1.48; 64.88 µg/mL	2.17; 145.56 ng/mL	1.71; 95.49 ng/mL
Intra-assay CV ^2^ %	3.40	4.72	1.92	1.94
QCs ^3^ Intra-assay CV%	4.26	3.05	2.38	1.63
Pool Intra-assay CV%	3.16	5.29	2.02	3.39
QCs Inter-assay CV%	4.98	9.65	6.77	16.63
Pool Inter-assay CV%	6.62	5.7	5.35	6.76

^1^ IQR, Interquartile Range; ^2^ CV, Coefficient of Variation; ^3^ QC, Quality Control.

## Data Availability

The data underlying this article will be shared on reasonable request to the head of the Division of Cancer Prevention and Genetics Bernardo Bonanni.

## Supplementary Materials

The following are available online at https://www.mdpi.com/article/10.3390/cancers13133303/s1, Table S1: Characteristics of breast cancers that occurred during follow-up; Figure S1: Disease-free survival curves for different quartiles of adiponectin and leptin.
