# Supplementary material for: A Novel Automated Immunoassay Platform to Evaluate the Association of Adiponectin and Leptin Levels with Breast Cancer Risk
Source: Cancers (Basel). 2021 Jun 30;13(13):3303. doi: 10.3390/cancers13133303 (PMC8268385; doi:10.3390/cancers13133303)
Supplement: Supplementary file 1 [file cancers-13-03303-s001.zip › cancers-1236013-supplementary.pdf]

Supplemental Table S1. Characteristics of breast cancers that occurred during follow-up

Patient	Disease status at baseline	Breast cancer type	Lymph-node involvement	Breast cancer grade	ER* status	PR* status	HER* status
1	HEALTHY*	DCIS	NO	2	positive	positive	negative
2	HEALTHY	DCIS	NO	2	unknown	unknown	unknown
3	HEALTHY	DCIS	NO	2	unknown	unknown	unknown
4	HEALTHY	INVASIVE	NO	2	positive	positive	negative
5	HEALTHY	INVASIVE	NO	3	negative	negative	positive
6	HEALTHY	INVASIVE	NO	2	positive	positive	negative
7	HEALTHY	INVASIVE	NO	2	positive	positive	negative
8	HEALTHY	INVASIVE	NO	2	positive	negative	negative
9	HEALTHY	INVASIVE	NO	3	positive	positive	unknown
10	HEALTHY	INVASIVE	NO	3	negative	negative	unknown
11	HEALTHY	INVASIVE	NO	1	positive	positive	unknown
12	HEALTHY	INVASIVE	NO	2	positive	positive	negative
13	HEALTHY	INVASIVE	YES	2	positive	positive	negative
14	HEALTHY	INVASIVE	YES	2	positive	positive	negative
15	DCIS*	DCIS	NO	2	unknown	unknown	unknown
16	DCIS	DCIS	unknown	2	negative	negative	positive
17	DCIS	INVASIVE	NO	2	positive	positive	unknown
18	DCIS	INVASIVE	NO	3	negative	negative	positive
19	DCIS	INVASIVE	NO	2	positive	negative	unknown
20[1]	DCIS	INVASIVE	NO	2	positive	positive	unknown
21[2]	DCIS	INVASIVE	unknown	2	positive	positive	negative

*ER, estrogen receptor; PR, progesteron receptor; HER, human epidermal growth factor receptor 2; HEALTHY: Subjects randomized in the Prevention IBIS-II Trial; DCIS, Ductal Carcinoma In Situ: Subjects randomized in the DCIS IBIS-II Trial

[1]Only leptin measurement available

[2]Only adiponectin measurement available

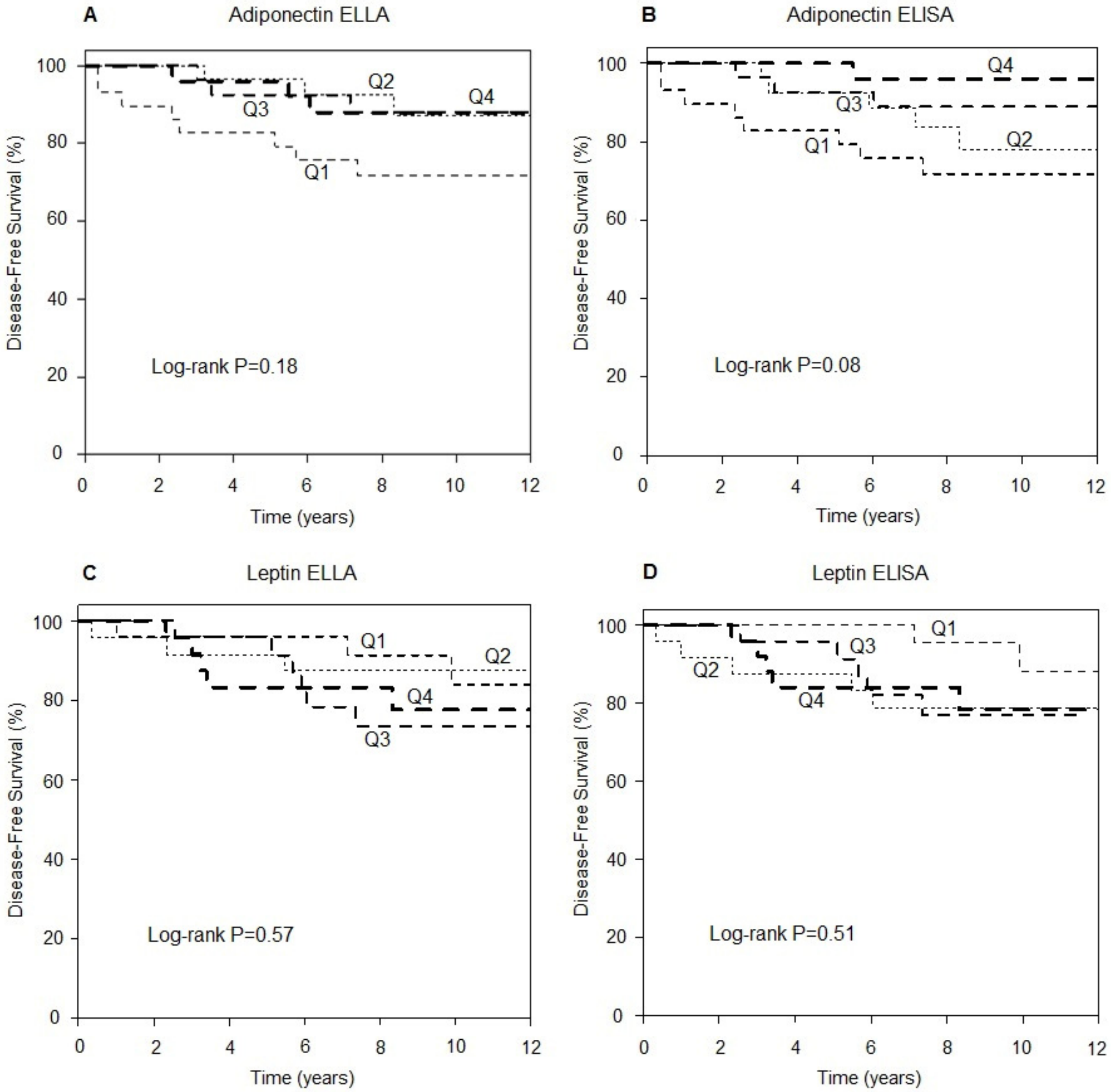

Supplemental Figure 1. Disease-free survival curves for different quartiles (Q1-4) of adiponectin measured with ELLA (A) or ELISA (B) and leptin measured with ELLA (C) or ELISA (D). Disease-free survival was calculated including subjects with resected DCIS and high risk healthy subjects.
